# Clinical Warburg Effect in a primary cutaneous lymphoma

**DOI:** 10.46989/001c.154811

**Published:** 2026-01-02

**Authors:** Francisco Martins, Guilherme Fontinha, José Magalhães, José Carlos Cardoso, Joana Calvão

**Affiliations:** 1 Dermatology Department, University Hospital, Coimbra’s Local Health Unit, Praceta Professor Mota Pinto, Celas, 3004-561, Coimbra, Portugal; 2 Anatomic Pathology Department, University Hospital, Coimbra’s Local Health Unit, Praceta Professor Mota Pinto, Celas, 3004-561, Coimbra, Portugal; 3 Internal Medicine Department, University Hospital, Coimbra’s Local Health Unit, Praceta Professor Mota Pinto, Celas, 3004-561, Coimbra, Portugal

**Keywords:** Lymphoma, Large B-Cell, Diffuse, Lactic Acidosis, Hypoglycemia, Clinical Warburg Effect, Primary Cutaneous Lymphoma

Primary cutaneous lymphomas are a subset of extranodal B- and T-cell malignancies characterized by skin-limited involvement at the time of diagnosis.^1^ Among these, primary cutaneous B-cell lymphomas (PCBCLs) comprise roughly 20% of cases and include both indolent subtypes - such as marginal zone lymphoma and follicle center lymphoma - and more aggressive variants – such as primary cutaneous diffuse large B-cell lymphoma, leg type (PCDLBCL-LT).^1–3^ When cases lack defining features, the diagnosis of primary cutaneous diffuse large B-cell lymphoma, not otherwise specified (PCDLBCL-NOS), is applied.^1^ Intravascular large B-cell lymphoma (IVLBCL) is a systemic lymphoma defined by tumor cell confinement to vessel lumina, but is sometimes considered alongside PCBCLs due to its frequent cutaneous presentation.^4,5^

The Warburg effect - a form of metabolic reprogramming in which tumor cells preferentially convert glucose to lactate despite adequate oxygen^6^ – is a hallmark of cancer. Though often undetectable, this shift may cause refractory hypoglycemia and type B lactic acidosis in high-grade malignancies - a life-threatening complication known as *clinical* Warburg effect.^7^ This is mechanistically distinct from tumor lysis syndrome (TLS), which results from acute cell death and release of intracellular contents.^8^ Unlike TLS, the Warburg effect is driven by sustained tumor metabolism and can occur in the absence of treatment.

We present a rare case of a primary cutaneous lymphoma manifesting with a clinical Warburg effect. An 84-year-old woman with no significant medical history presented with four weeks of fatigue and a rapidly progressive eruption of indurated, violaceous nodules over the face, cervicothoracic region, and arms. Laboratory evaluation revealed elevated lactate dehydrogenase (1786 IU/L) and β2-microglobulin (8.94 mg/L), with otherwise unremarkable hematologic and biochemical parameters.

During hospitalization, she developed recurrent hypoglycemia, initially diet-responsive but later requiring IV glucose. On day 5, she became acutely disoriented and tachypneic. Arterial blood gas analysis revealed profound lactic acidosis: pH 6.90, bicarbonate 4.9 mmol/L, and serum lactate 21.1 mmol/L, with preserved oxygenation (pO₂ 88 mmHg). Electrolytes, including potassium and phosphate, remained within normal limits. Despite prompt administration of intravenous bicarbonate and glucose, the patient’s condition deteriorated rapidly, and she died within one hour.

A complete autopsy was performed to clarify the cause of rapid clinical deterioration. Gross examination revealed over 50 violaceous nodules distributed across the trunk, face, and upper limbs, with no significant abnormalities identified in internal organs. Histopathologic analysis of a representative skin lesion demonstrated a dense dermal and subcutaneous infiltrate (***Figure 1A***) composed of large atypical lymphoid cells with features of centroblasts and immunoblasts (***Figure 1B***). Immunohistochemistry showed positivity for CD20 and BCL2, and negativity for CD10, BCL6, and MUM1. The Ki-67 proliferation index was approximately 70%. Fluorescence in situ hybridization (FISH) revealed no MYC rearrangements. Flow cytometry of the skin lesion (performed antemortem) demonstrated expression of CXCR5.

**Figure 1.**
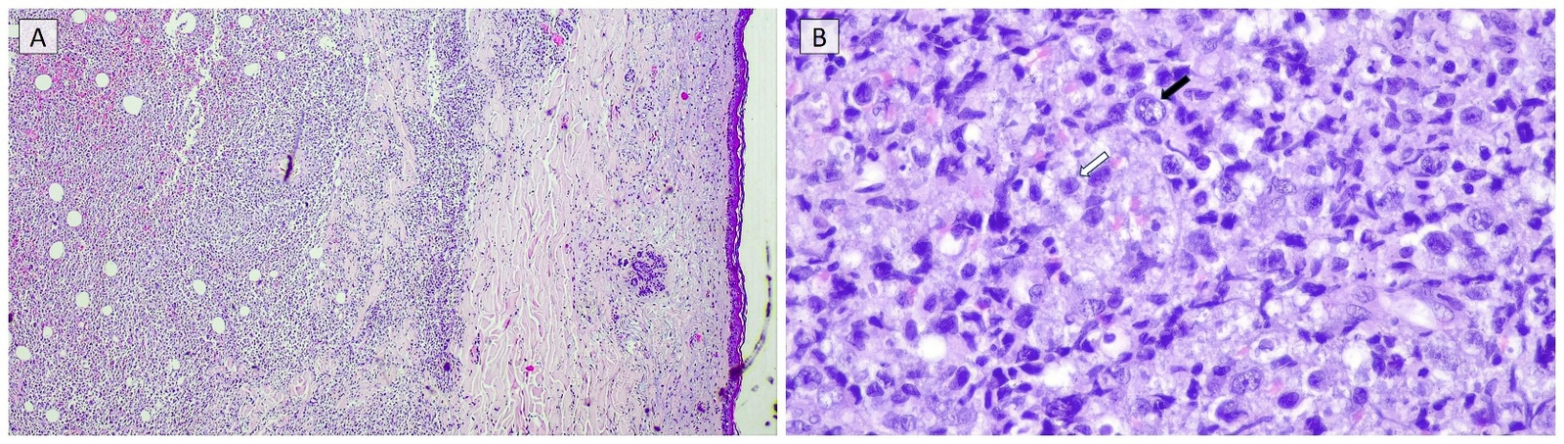
Histopathology of a cutaneous nodule. (A) Dense and nodular lymphoid infiltrate occupying the dermis (H&E, original magnification ×40). (B) Higher magnification revealing large atypical lymphoid cells with immunoblastic (white arrow) and centroblastic features (black arrow), outside the lumen of vessels (H&E, original magnification ×200).

Microscopic examination of internal organs - including the spleen, liver, lungs, ovaries, uterus, bladder, and colon - revealed exclusively intravascular localization of neoplastic cells without parenchymal infiltration or mass formation (***Figure 2, A-D***). No lymph node involvement was observed, and the bone marrow showed only focal intravascular tumor cell aggregates. Outside the skin, the lymphoma was confined entirely to the lumina of vessels.

**Figure 2.**
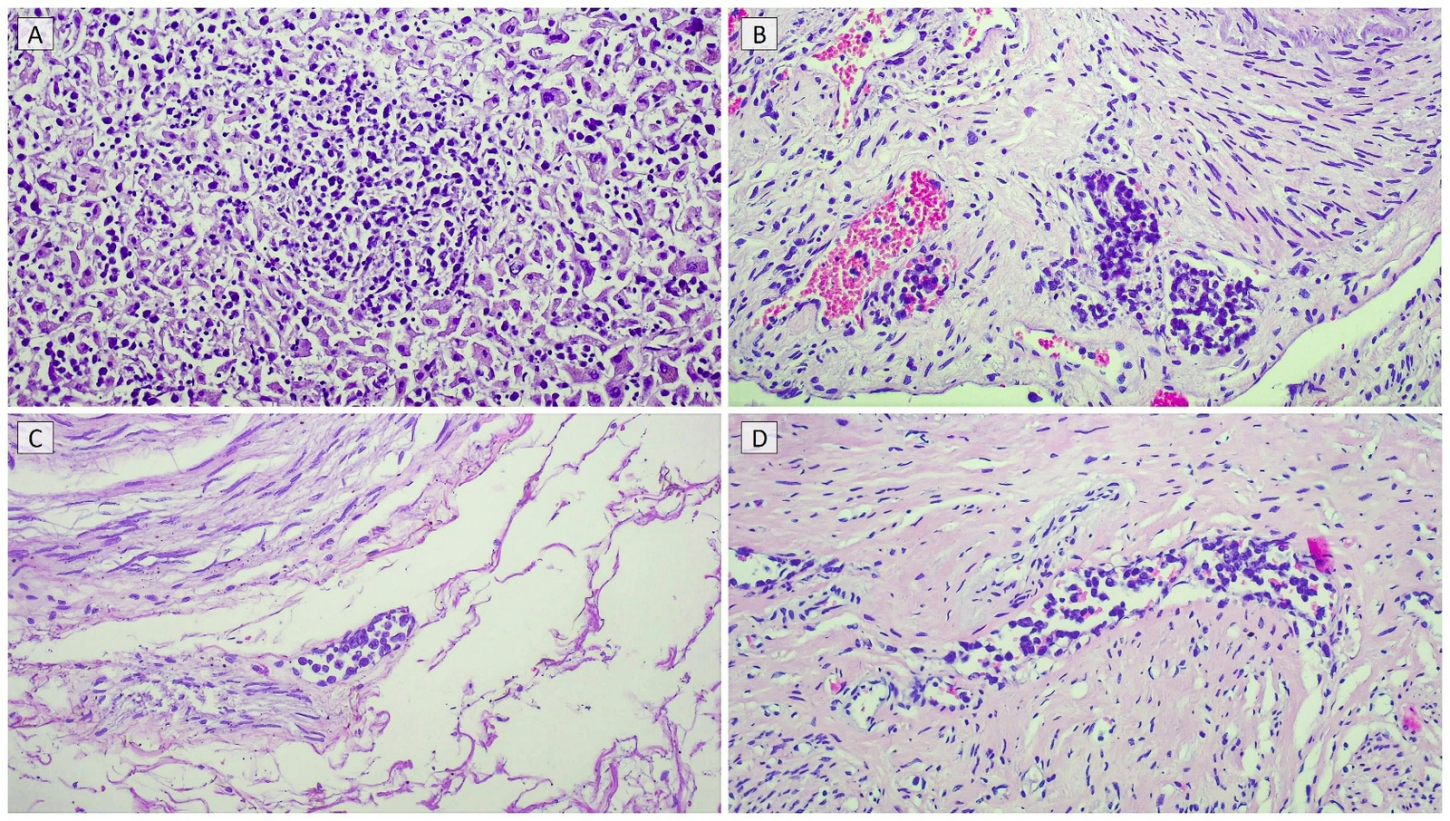
Autopsy specimens showing intravascular dissemination of lymphoma cells within (A) hepatic sinusoids, (B) ovarian vessels, (C) colonic mucosa, and (D) uterine vessels, without parenchymal infiltration (H&E, ×100).

These findings were consistent with the diagnosis of an extranodal diffuse large B-cell lymphoma with both cutaneous mass-forming and systemic intravascular involvement. Death was attributed to fulminant type B lactic acidosis and hypoglycemia resulting from tumor-driven glycolysis, despite preserved oxygenation - a clinical Warburg effect. Notably, repeated glucose infusions, intended to correct hypoglycemia, likely exacerbated lactate production, paradoxically accelerating clinical deterioration.

Most diffuse large B-cell lymphomas involve lymph nodes, with 30% being primary extranodal (such as primary cutaneous DLBCL).^9^ In this case, the diagnostic challenge lay in determining the sequence of events: whether this represented IVLBCL with secondary cutaneous infiltration, or a primary cutaneous lymphoma (leg-type or NOS) that later disseminated intravascularly.

The presence of intravascular cells and a fulminant clinical course initially raised suspicion for IVLBCL, but multiple findings argued strongly against it: (1) the skin lesions were entirely extravascular, in contrast to the hallmark intravascular growth pattern of IVLBCL, (2) the tumor expressed CXCR5 (a chemokine receptor that suggests preserved tissue migration and is typically absent in IVLBCL), (3) there was no central nervous system involvement and (4) MUM1 was negative.^10–13^

As the entirety of mass-forming disease was confined to the skin, the most consistent diagnosis was PCDLBCL-NOS with secondary intravascular dissemination (***Figure 3***). While PCDLBCL-LT may occasionally present off the legs and shares features such as BCL2 expression and high Ki-67, our case lacked its typical MUM1 and BCL6 co-expression.^14,15^ PCDLBCL-NOS represents a heterogeneous category defined by exclusion, encompassing uncommon presentations such as the one described here.^1,2^

**Figure 3.**
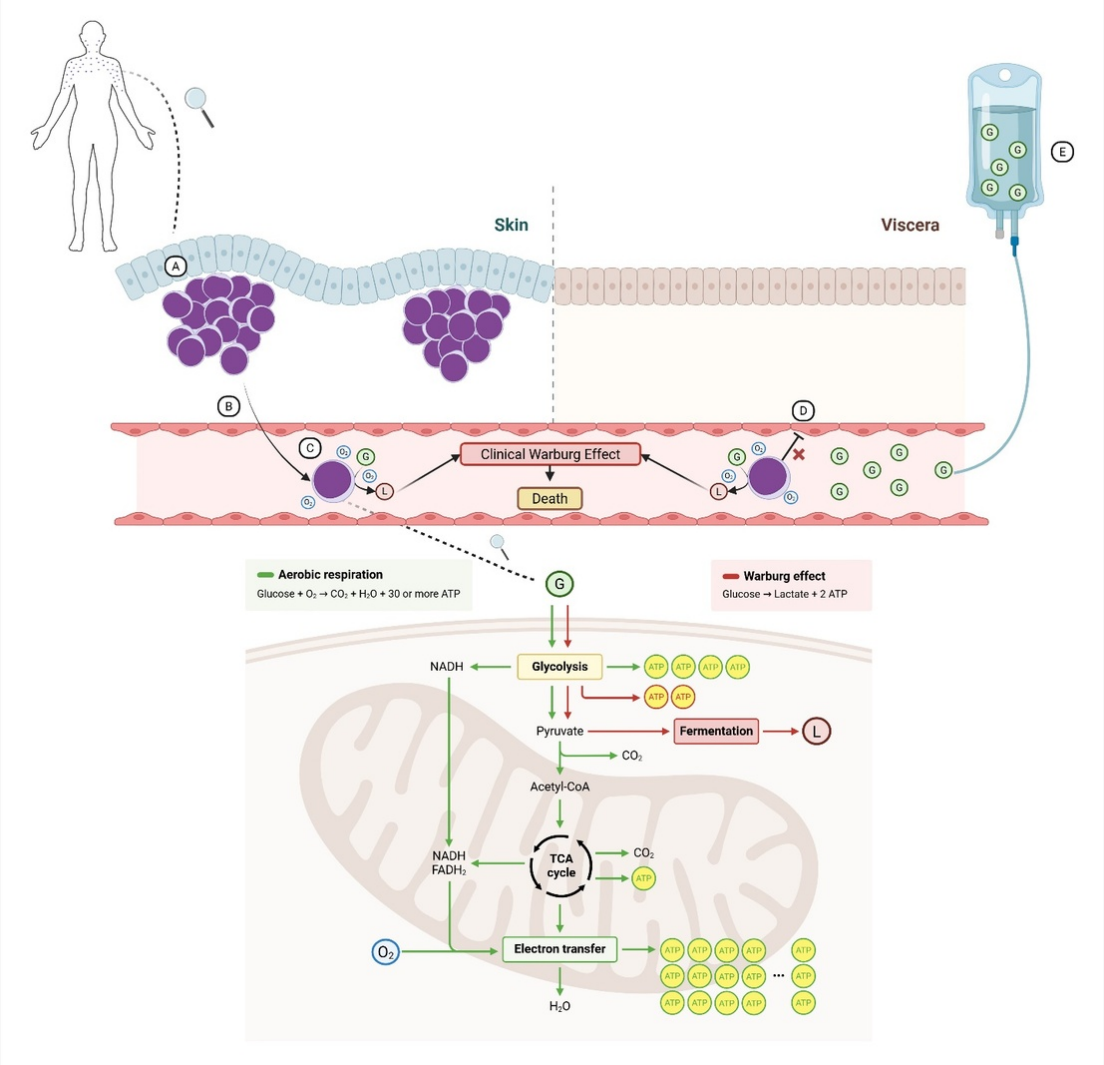
Proposed mechanism of intravascular dissemination in primary cutaneous diffuse large B-cell lymphoma, not otherwise specified (PCDLBCL-NOS), leading to a clinical Warburg effect. (A) The lymphoma initially presented as cutaneous nodules predominantly involving the face, cervicothoracic region and upper limbs. (B) Neoplastic cells disseminated via the vasculature without infiltrating parenchymal organs. (C) Despite adequate oxygen availability, lymphoma cells preferentially utilized aerobic glycolysis - a metabolically inefficient pathway that generates only 2 ATP per glucose molecule and lactate as a byproduct. (D) Beyond the skin, the exclusive intravascular localization of highly glycolytic tumor cells led to marked lactate accumulation, manifesting as a clinical Warburg effect with rapid deterioration and death. (E) Administration of intravenous glucose during supportive care may have paradoxically exacerbated lactic acidosis by fueling the continued glycolytic metabolism of circulating lymphoma cells. Legend: ATP – adenosine triphosphate; G – glucose; L – lactate; TCA – tricarboxylic acid. Created with BioRender.

The last tension point lay in determining whether the presence of intravascular tumor cells could still be compatible with the diagnosis of a primary cutaneous lymphoma. Increasing evidence suggests this might be the case: in cutaneous T-cell lymphomas, circulating clones have been shown to reseed distant skin sites,^16^ and in PCBCLs, detection of circulating tumor DNA indicates that cutaneous disease coexists with low-level systemic dissemination.^17^

Our case of PCDLBCL-NOS complicated by a clinical Warburg effect thus aligns with an evolving view of primary cutaneous lymphomas as biologically systemic diseases, where skin predominance reflects tropism rather than true compartmental restriction.

## Authors’ Contribution

Conceptualization and writing original draft preparation: Francisco Martins; Review and editing: Guilherme Fontinha, José Magalhães, José Carlos Cardoso, Joana Calvão; Supervision: Joana Calvão.

## Competition of Interest – COPE

No competing interests were disclosed.

## Ethical Conduct Approval – Helsinki – IACUC

Informed consent was obtained and all procedures in this work were in accordance with current institutional ethical standards.

## Informed Consent Statement

All authors and institutions have confirmed this manuscript for publication.

## Data Availability

All are available upon reasonable request.
